# Silicon Promotes Adventitious Shoot Regeneration and Enhances Salinity Tolerance of *Ajuga multiflora* Bunge by Altering Activity of Antioxidant Enzyme

**DOI:** 10.1155/2014/521703

**Published:** 2014-01-09

**Authors:** Iyyakkannu Sivanesan, Byoung Ryong Jeong

**Affiliations:** ^1^Institute of Agriculture and Life Science, Gyeongsang National University, Jinju 660-701, Republic of Korea; ^2^Research Institute of Life Science, Gyeongsang National University, Jinju 660-701, Republic of Korea; ^3^Department of Horticulture, Division of Applied Life Science (BK21 Plus), Graduate School, Gyeongsang National University, Jinju 660-701, Republic of Korea

## Abstract

We investigated the effect of Si concentration on shoot regeneration and salinity tolerance of *Ajuga multiflora*. Addition of Si to the shoot induction medium significantly increased the frequency of shoot induction. The average number of shoots regenerated per explant decreased on the medium containing NaCl alone, while there was less decrease when the shoot induction medium was supplemented with both NaCl and Si. The shoot induction percentage increased linearly with increasing concentration of Si in the NaCl containing medium. Addition of Si to the shoot induction medium significantly increased SOD, POD, APX, and CAT activity in regenerated shoot buds as compared with the control. The inclusion of Si to the NaCl containing medium significantly increased the SOD activity in leaves and roots, while it decreased POD, APX, and CAT activity in both organs. Scanning electron microscopic analysis showed that there are no distinct differences in the structure of stomata between the control and Si-treated plants. However, NaCl treatment significantly affected the structure and number of stomata as compared to the control. Wavelength dispersive X-ray analysis confirmed the high Si deposition in trichomes of plants grown in the Si containing medium but not in plants grown in the medium without Si.

## 1. Introduction

Silicon (Si) is most abundant mineral element both on the surface of the earth's crust and in the soil. In soil solutions, Si is found mainly as silicic acid which is taken up by plant roots. Though Si is present in most of the soil grown plants, but it is not considered to be an essential element for plant growth and development [1]. However, Si deficiency symptoms were observed in cucumber, sugarcane, and tomato [2]. Recent studies show the beneficial effects of Si on growth, development, and yield of plants [3–5]. Further, Si plays an important role in enhancing the resistance of plants to abiotic and biotic stresses such as drought, frost, metal toxicity, nutrient imbalance, salinity, and diseases [6]. The ameliorative effect of Si on plants suffering from abiotic stresses often occurs through counteracting oxidative stress by modulating antioxidant enzymes [7].

*In vitro* culture technique is often used for mass propagation of economically important plant species. *In vitro* morphogenetic response of plant species mainly depends on the addition of plant growth regulators to the culture medium [8]. Similarly, antioxidant enzyme activity has also marked effect on the growth and morphogenesis [9]. However, several physiological problems associated with the micropropagation such as browning, hyperhydricity, and shoot-tip necrosis are often encountered during the different stages of micropropagation [10]. These problems are mainly associated with culture environmental and nutritional factors. Further, *in vitro* regenerated plantlets often exhibit low survival rate due to transplantation shock caused by abiotic and biotic stresses. The application of Si has been shown to be beneficial for several plants particularly when those plants are exposed to abiotic and biotic stresses [6, 7]. Addition of Si to the culture medium reduced hyperhydricity in *Cotoneaster wilsonii* [11] and *Ornithogalum dubium* [12]. The addition of Si to the culture medium has been proven to be advantageous for callus induction and plant regeneration in rice [13]. Recently, Máthé et al. [14] reported that Si has a significant effect on somatic embryo development, plant, and root morphogenesis in reed. The Si treatment reduced incidence of hyperhydricity by decreasing the accumulation of hydrogen peroxide and malondialdehyde and lowering the activity of enzymes ascorbate oxidase and glutathione reductase [11, 12]. However, the mode of action of Si on shoot regeneration is not reported.

Soil salinity is one of the major abiotic stresses limiting growth and development of most plant species. In floricultural crops salinity affects growth, plant, and flower marketable quality, including visual injury, flower distortion, and reduced stem length [15]. Little information is available on salt tolerance of floricultural crops [16]. Thus, it is important to determine salt tolerance of bedding plants to minimize potential salt injuries. The determination of optimal Si concentration for salt tolerance of plants under *in vivo* conditions poses difficulties because of the complex interactions existing between the plant and different soil components [17]. Further, Si is a ubiquitous contaminant. *In vitro* culture is an ideal system for screening of salinity tolerance competence in plants as it can be carried out under controlled conditions with known amount of Si and other nutrients.

The genus *Ajuga, *belonging to the mint family (Lamiaceae), comprises about 301 species and occurs in the cooler parts of Europe, Asia, Africa, and Australia. The *Ajuga* species grow to 5–50 cm tall, with opposite leaves, which are attractive. The flowers are two-lipped and tubular and mostly blue, purple, or yellow in color. Many *Ajuga *species are used in horticulture as groundcover or border and in rock gardens but some are regarded as weeds [18]. *Ajuga multiflora* Bunge is a perennial herb 8–13 cm tall, widely distributed in Korea, north-eastern part of China, and Russia. It is cultivated as an ornamental groundcover in Korea and also used in a traditional medicine for the treatment of fever. It is reported to contain several phytoecdysteroids [19]. The plant is typically propagated by division of rhizomes. In order to provide enough plant material for commercial exploitation, the propagation of the plant using rhizome is not sufficient. *In vitro* propagation technique is being used widely for large-scale production of many plant species. So far, only few reports are available on *in vitro* propagation of *Ajuga* species [20–22]. The objective of this study was to determine the effect of Si on shoot regeneration and salt tolerance of *A. multiflora*.

## 2. Materials and Methods

### 2.1. Plant Materials and Surface Sterilization

Leaves with petioles were excised from greenhouse-grown plants, washed initially under running tap water for 30 min, and then washed thoroughly in distilled water. The explants were surface-sterilized in 70% (v/v) ethanol for 60 sec, 1.5% (v/v) sodium hypochlorite for 15 min, and 0.1% (w/v) HgCl_2_ for 5 min. Each treatment was followed by 5 washes with sterile distilled water [22].

### 2.2. Effect of N^6^-(2-isoPentenyl)adenine (2iP) and Indole-3-Acetic Acid (IAA) on Adventitious Shoot Regeneration

Leaf and petiole explants (0.5–1.0 cm) were prepared and inoculated on Murashige and Skoog (MS) medium containing 4.4, 8.8, or 12.2 *μ*M 2iP along with 5.7 *μ*M IAA for shoot bud induction. The medium consisted of MS salts and vitamins [23] supplemented with 3% (w/v) sucrose and solidified with 0.8% (w/v) agar. The pH of the medium was adjusted to 5.8 before autoclaving. The cultures were maintained at 25 ± 1°C under a 16 h photoperiod with 45 *μ*mol m^−2^ s^−1^ photosynthetic photon flux density (PPFD). The number of explants initiating shoot buds and the average number of shoot buds per explant were recorded after 35 days.

### 2.3. Effect of Si and NaCl on Adventitious Shoot Regeneration, Shoot Growth, and Rooting

Leaf and petiole explants were cultured on shoot induction (MS + 12.2 *μ*M 2iP + 5.7 *μ*M IAA) medium containing 0, 1.8, 3.6, or 7.2 mM Si alone or in combination with 50 mM NaCl. Regenerated shoots were excised from explants and transferred to the respective medium without plant growth regulators and cultured for five weeks to induce the growth of shoots and roots.

### 2.4. Estimation of Antioxidant Enzymes

Regenerated shoot buds, leaf, and root tissue (0.1 g) were homogenized in 50 mM phosphate buffer (pH 7.0) containing 1.0 mM EDTA, 0.05% Triton X-100, 1.0 mM polyvinylpyrrolidone (PVP), and 1.0 mM ascorbate. The homogenate was centrifuged at 13,000 rpm at 4°C. The supernatant was used to estimate the activities of antioxidant enzymes. Protein content was estimated by the method of Lowry et al. [24]. Superoxide dismutase (SOD) activity was assayed by measuring its ability to inhibit the photochemical reduction of nitroblue tetrazolium (NBT) according to the method of Beauchamp and Fridovich [25]. Peroxidase (POD) activity was measured by following the procedure of Sadasivam and Manickam [26]. Ascorbate peroxidase (APX) activity was estimated according to the protocol described by Chen and Asada [27]. Catalase (CAT) activity was determined based on the method by Aebi [28].

### 2.5. Scanning Electron Microscopy (SEM) and Wavelength Dispersive X-Ray Analysis (WDXA)

Leaves were separated from the Si and NaCl treatments. Leaf samples were cut into about 0.5 mm^2^ size and fixed in 3.0% (v/v) glutaraldehyde (pH 7.5) overnight. Samples were washed three times with 0.1 M PBS buffer (137 mM NaCl + 2.7 mM KCl + 43 mM Na_2_HPO_4_ + 1.4 mM KH_2_PO_4_) and then fixed in 1.0% (w/v) osmium tetroxide (pH 7.2) for 2 h at 4°C. The samples were washed four times with a PBS buffer, dehydrated through an ethanol series, and dried with a critical point dryer (CPD2, Pelco, CA, USA). Dried samples were positioned on aluminum stubs with double stick tape prior to gold coating in a sputter coater (SC7640, Polaron, Sussex, UK). The deposition of Si, K, and Na in leaf samples was analyzed by a point analysis with wavelength dispersive spectrometer (JXA-8100, Jeol, Tokyo, Japan) combined with a SEM (LEO-435VP, Zesis, Jena, Germany) at 15 kV.

### 2.6. Statistical Analysis

The experiments were repeated thrice and 30 explants or shoot buds were used per treatment. Data were subjected to analysis of variance (ANOVA) by using SAS program (Release 9.1, SAS Institute, NC, USA), and Duncan's multiple range test was used to assess significant differences between mean values.

## 3. Results and Discussion

### 3.1. Effect of 2iP and IAA on Adventitious Shoot Regeneration

**Figure 1:**
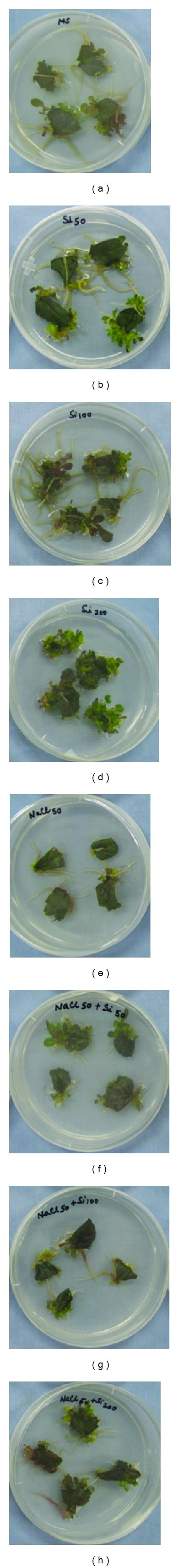
Effect of silicon and NaCl on adventitious shoot regeneration from leaf explants of *A. multiflora*. (a) MS medium with 12.2 *μ*M 2iP and 5.7 *μ*M IAA (SIM), (b) SIM + 1.8 mM Si, (c) SIM + 3.6 mM Si, (d) SIM + 7.2 mM Si, (e) SIM + 50 mM NaCl, (f) SIM + 50 mM NaCl + 1.8 mM Si, (g) SIM + 50 mM NaCl + 3.6 mM Si, and (h) SIM + 50 mM NaCl + 7.2 mM Si.

**Figure 2:**
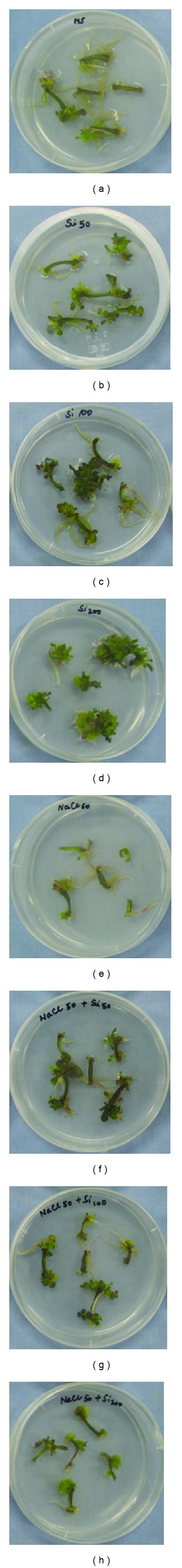
Effect of silicon and NaCl on adventitious shoot regeneration from leaf explants of *A. multiflora*. (a) MS medium with 12.2 *μ*M 2iP and 5.7 *μ*M IAA (SIM), (b) SIM + 1.8 mM Si, (c) SIM + 3.6 mM Si, (d) SIM + 7.2 mM Si, (e) SIM + 50 mM NaCl, (f) SIM + 50 mM NaCl + 1.8 mM Si, (g) SIM + 50 mM NaCl + 3.6 mM Si, and (h) SIM + 50 mM NaCl + 7.2 mM Si.

Several ornamental plants are at present propagated *in vitro* by direct adventitious shoot regeneration. The surface sterilization method yielded 98% aseptic explants. Cytokinins are very effective in promoting adventitious shoot formation. However, both leaf and petiole explants did not respond well when 2iP alone was used [22]. In many plants, combination of auxin and cytokinin produced more shoots than cytokinin alone [8, 11]. Thus, in this study we investigated the effect of combination of 2iP and IAA on adventitious shoot regeneration from leaf and petiole explants of *A. multiflora*. The explants cultured on plant growth regulators free MS medium did not develop shoot buds. When the MS medium was supplemented with 2iP and IAA both leaf and petiole explants developed shoot buds. Adventitious shoot buds were induced from the cut ends of both explants within 14 days of culture. An increase in the concentration of 2iP with constant IAA increased the percentage of shoot induction and number of shoots in both explants (Table 1 and Figures 1(a) and 2(a)). The greatest percentage of shoot induction with average number of shoots per explants was obtained on MS medium containing 12.2 *μ*M 2iP and 5.7 *μ*M IAA. Similar result was also observed in* Rhododendron keiskei *var. *hypoglaucum *[11].

### 3.2. Effect of Si and NaCl on Adventitious Shoot Regeneration, Shoot Growth, and Rooting

Addition of Si to the shoot induction medium significantly increased both the frequency of shoot induction and average number of shoots per explant. Si has been proven to be effective for shoot regeneration in reed [14] and rice [13]. Increasing concentration of Si in the shoot induction medium significantly increased the average number of shoots per explant. Among the various concentrations tested, 7.2 mM Si was found to be the best for adventitious shoot induction in both explants. When the explants were cultured on the shoot induction medium supplemented with 0–7.2 mM Si, the average number of shoot buds produced per explant ranged 8.5–22.7 in leaf (Table 2 and Figures 1(a)–1(d)) and 6.2–18.4 in petiole (Table 2 and Figures 2(a)–2(d)). Adventitious shoot formation was obtained with a frequency of 73.3–100% in leaf and 76.6–100% in petiole explants of *A. multiflora *cultured on the shoot induction medium supplemented with 0–7.2 mM Si. Among the two explants tested, leaf explant developed the greatest number of shoots than petiole explant. The highest frequency of shoot induction (100%) with the average number of 22.7 shoots per leaf explant and 18.4 shoots per petiole explant was obtained on the shoot induction medium supplemented with 7.2 mM Si. Gibberellins (GA) are plant growth regulators that play a pivotal role in the growth and development of plants. Si treatment increased GA_1_ and GA_20_ levels in rice cultivars [29]. The stimulating effect of GA_3_ on shoot induction has been reported in apple [30], *Cephaelis ipecacuanha* [31], and citrus [32]. Thus, the positive effect of Si on shoot induction may be due to altered endogenous level of GA (Table 2).

A preliminary experiment was conducted to test the effect of various concentrations of NaCl (0, 25, 50, or 100 mM) on shoot regeneration of leaf and petiole explants of *A. multiflora*. Shoot regeneration was completely inhibited at 100 mM NaCl. Addition of 50 mM NaCl to the shoot induction medium significantly decreased the percentage of shoot induction and number of shoots in both explants as compared with the control (Table 2 and Figures 1(e) and 2(e)). Thus, for further studies 50 mM NaCl was used. The average number of shoots regenerated per explant decreased on the medium containing NaCl alone, while there was less decrease when the shoot induction medium was supplemented with both NaCl and Si (Table 2 and Figures 1(e)–1(h) and Figures 2(e)–2(h)). The *in vitro* morphogenetic response of plants also depends on the medium composition. The high concentrations of Cl^−^ and Na^+^ ions led to ion imbalance in the culture medium and thereby reduced the number of regenerated shoots per explant. Si plays an important role under conditions of nutrient imbalance [6]. The shoot induction percentage increased linearly with increasing concentration of Si in the NaCl containing medium. In both explants, the highest frequency of shoot induction (100%) was observed on the shoot induction medium containing 50 mM NaCl and 7.2 mM Si. The highest number of 10.7 shoots per leaf explant and 8.7 shoots per petiole explant was obtained on the shoot induction medium containing 50 mM NaCl with 7.2 or 3.6 mM Si, respectively.

**Figure 3:**
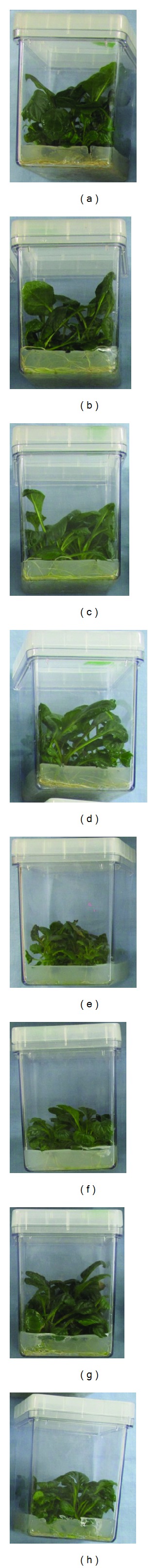
Effect of silicon and NaCl on shoot growth and rooting of *A. multiflora*. (a) MS medium, (b) MS + 1.8 mM Si, (c) MS + 3.6 mM Si, (d) MS + 7.2 mM Si, (e) MS + 50 mM NaCl, (f) MS + 50 mM NaCl + 1.8 mM Si, (g) MS + 50 mM NaCl + 3.6 mM Si, and (h) MS + 50 mM NaCl + 7.2 mM Si.

The regenerated shoots were separated from the explants and cultured on the plant growth regulators free MS medium for further shoot growth and rooting. Addition of Si significantly increased plant height, chlorophyll content, root length, and fresh and dry weights of shoot and root as compared with the control. Inclusion of 1.8 or 3.6 mM Si to the MS medium increased the plant height, but increasing the concentration of Si to 7.2 mM led to shorter plant as compared with the control (Table 3 and Figures 3(a)–3(d)). The average number of leaves per shoot decreased linearly with increasing concentration of Si. The greatest chlorophyll content (3.75 *μ*g mg^−1^ fw) was recorded on the MS medium supplemented with 3.6 mM Si. However, increasing Si concentration above 3.6 mM had a negative effect on chlorophyll content. Root length significantly increased with increasing concentration of Si in the culture medium. The greatest root length (15.4 cm) was obtained on the MS medium containing 7.2 mM Si. Addition of Si significantly increased fresh and dry weights of shoot and root as compared with the control. The highest fresh and dry weights of shoot and root were obtained on the MS medium containing 3.6 mM Si. However, addition of 50 mM NaCl significantly reduced the growth traits of *A. multiflora* as compared with the control (Figure 3(e)). Si has been shown to be effective in alleviating salt stress in various plants. In the present study, Si inclusion promoted growth traits on the medium containing 50 mM NaCl (Table 3 and Figures 3(g) and 3(h)). The greatest plant height, number of leaves, chlorophyll content, root length, and fresh and dry weights of shoot and root were obtained on the shoot induction medium containing 50 mM NaCl and 3.6 mM Si (Table 3). Similarly, Si treatment enhanced the growth traits of many salt-stressed plants [6, 7].

### 3.3. Estimation of Antioxidant Enzymes

Addition of Si to the culture medium promoted shoot regeneration; however, the mechanism of Si action is not known. In general, Si treatment significantly affected the antioxidant enzyme activities in many plants [6, 7]. Antioxidant enzymes enhanced the growth and morphogenesis in many plants. Thus, we investigated the effect of Si on antioxidant enzyme activities of *A. multiflora*. Addition of Si to the shoot induction medium significantly increased SOD, POD, APX, and CAT activity in regenerated shoot buds as compared with the control (Table 4). Similarly, the activity of antioxidant enzymes increased during organogenesis in *Brassica rapa *var. *turnip *[33], *Caladium bicolor *[34], *Crocus sativus *[35], *Piper nigrum *[9], and plum [36]. The activities of SOD and POD increased significantly with increasing concentration of Si in the shoot induction medium. The activity of CAT was significantly higher on the shoot induction medium containing 3.6 mM Si. Inclusion of 1.8 mM Si did not change the APX activity as compared with the control. However, the activity of APX was significantly increased by 3.8 or 7.8 mM Si treatment (Table 4). Thus, apparently Si promoted shoot regeneration of *A. multiflora *by altering activity of antioxidant enzymes.

We also estimated the antioxidant enzymes activities in leaves and roots of plantlets grown in the medium containing Si and NaCl alone or in combination of Si and NaCl. Inclusion of Si to the MS medium increased SOD in both leaves and roots as compared with the control. In contrast, addition of NaCl to the MS medium significantly decreased SOD activity in both leaves and roots as compared with the control. However, inclusion of Si to the NaCl containing medium significantly increased the SOD activity in both organs. Similarly, Si treatment increased leaf and root SOD activity in barley under NaCl stress *in vivo* [37]. The activity of SOD was significantly higher in both organs when the MS medium was supplemented with 3.6 mM Si. Si treatment significantly decreased POD activity in leaves, whereas inclusion of Si to the MS medium significantly increased POD activity in roots (Table 5). Addition of NaCl to the MS medium significantly increased POD activity in leaves, whereas it did not affect POD activity in roots as compared with the control. In leaves, the activity of POD was decreased on the culture medium containing both NaCl and Si. When the NaCl containing medium was supplemented with 3.6 or 7.2 mM Si the activity of POD in roots was significantly increased or decreased, respectively. In both leaves and roots, the activity of APX increased linearly with increasing concentrations of Si in the MS medium. Addition of NaCl to the MS medium significantly increased the activity of APX in both leaves and roots as compared with the control and other treatments. Inclusion of Si to the NaCl containing medium significantly decreased the APX activity in both organs. Similarly, the activity of CAT was significantly higher when the MS medium was supplemented with 50 mM NaCl. However, addition of Si decreased the CAT activity in both organs as compared with the 50 mM NaCl treatment (Table 5). Similar results were also reported in lettuce [38] and tomato [39]. In contrast, addition of Si increased the activities of CAT and APX in salt-stressed barley [40] and cucumber [41].

### 3.4. SEM and WDXA

**Figure 4:**
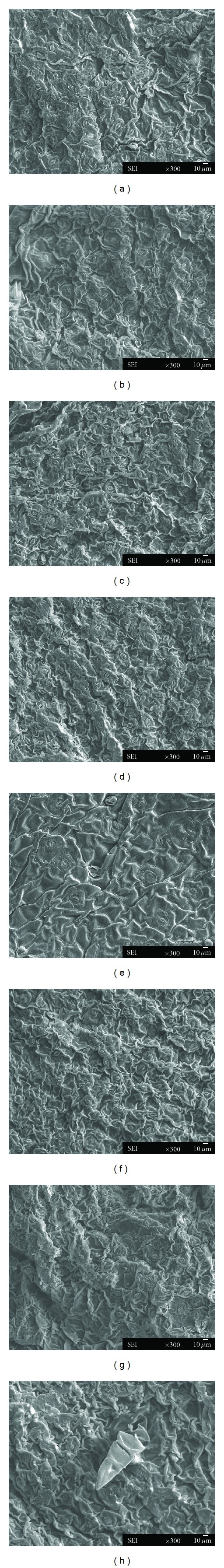
Scanning electron microscopic analysis of *A. multiflora* leaves treated with silicon and NaCl. (a) MS medium, (b) MS + 1.8 mM Si, (c) MS + 3.6 mM Si, (d) MS + 7.2 mM Si, (e) MS + 50 mM NaCl, (f) MS + 50 mM NaCl + 1.8 mM Si, (g) MS + 50 mM NaCl + 3.6 mM Si, and (h) MS + 50 mM NaCl + 7.2 mM Si.

**Figure 5:**
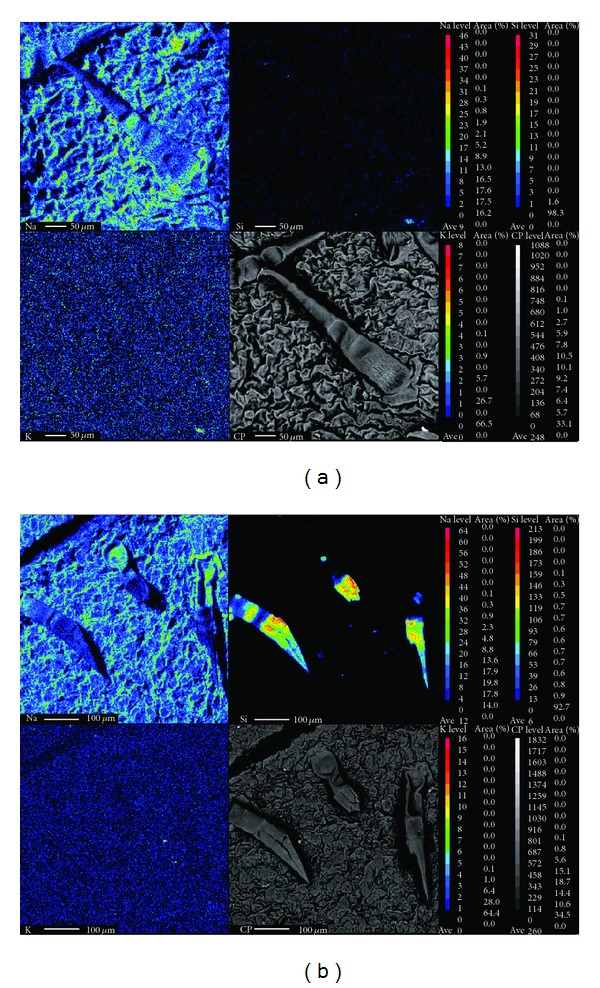
Wavelength dispersive X-ray analysis of Si-treated and nontreated leaves of *A. multiflora*. (a) MS medium and (b) MS + Si.

**Figure 6:**
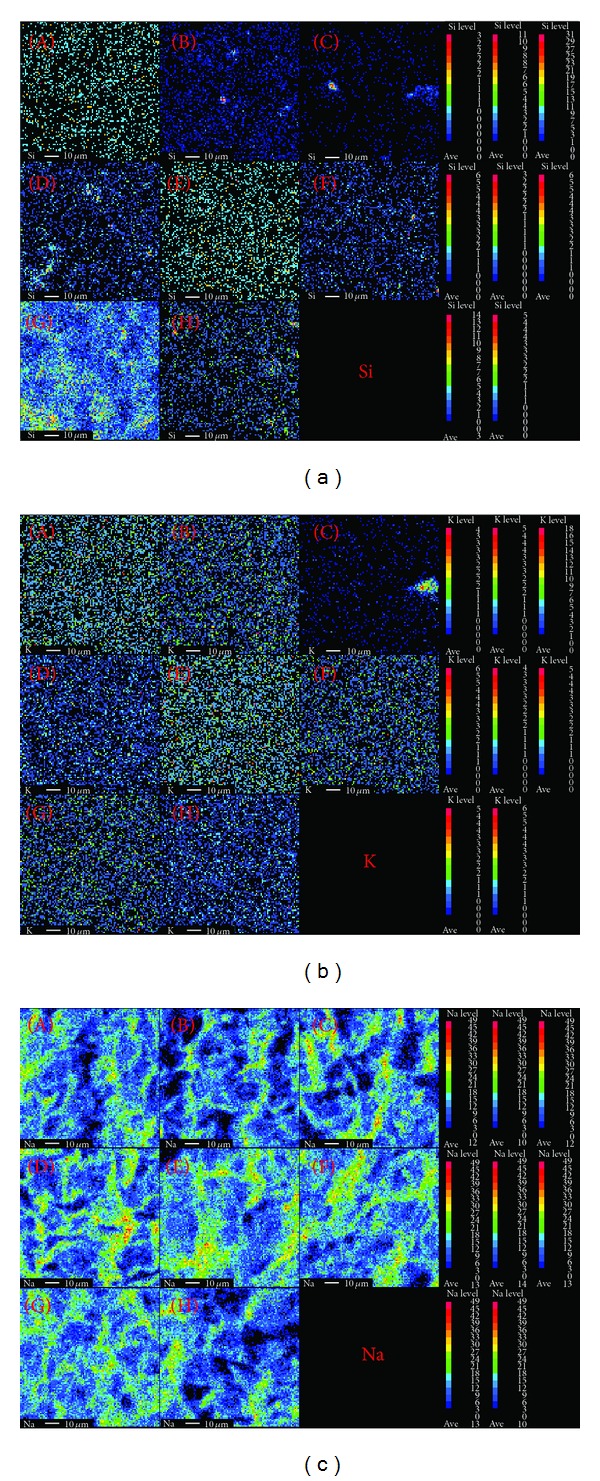
Wavelength dispersive X-ray analysis of Si, K, and Na in leaves of *A. multiflora* treated with silicon and NaCl. (A) MS medium, (B) MS + 1.8 mM Si, (C) MS + 3.6 mM Si, (D) MS + 7.2 mM Si, (E) MS + 50 mM NaCl, (F) MS + 50 mM NaCl + 1.8 mM Si, (G) MS + 50 mM NaCl + 3.6 mM Si, and (H) MS + 50 mM NaCl + 7.2 mM Si.

The SEM analysis showed that there is no distinct difference in the structure of stomata between the control and Si-treated plants (Figures 4(a)–4(d)). Similar result was observed in maize [42] on *in vivo* Si treatment. In contrast, addition of Si to the MS medium altered the stomata structure in begonia and pansy [43]. The functions, structure, and number of stomata were adversely affected by salinity. In the present study, NaCl treatment significantly affected the structure and number of stomata as compared to the control (Figure 4(e)). The leaf surface and stomata were normal when the NaCl containing medium was supplemented with 1.8–7.2 mM Si, but changes in the leaf surface were observed among the treatments (Figures 4(f)–4(h)). It has been reported that in several plant species Si mostly accumulates in leaves than other organs. In leaves, Si is accumulated mainly in epidermal cells, stomata, and trichomes [44, 45]. In the present study, WDXA confirmed the high Si deposition in trichomes of plants grown in the Si containing medium but not in plants grown in the medium without Si (Figures 5(a) and 5(b)). We also examined Si, K, and Na deposition in stomata of plants grown in the medium containing Si and NaCl alone or in combination. The Si deposition was observed in leaf surface of plants grown in the Si or Si + NaCl containing medium but not in the Si deprived or NaCl containing medium. The 3.6 mM Si treatment significantly increased the K deposition as compared with the control and other treatments. The deposition of Na was increased when the MS medium was supplemented with 50 mM NaCl. However, addition of Si to the NaCl containing medium significantly reduced the deposition of Na in a dose-dependent manner (Figures 6(A)–6(H)). Similarly, concentration of Na in shoots of barley [46], *Phaseolus vulgaris *[47], and rice [48] was decreased by inclusion of Si.

In conclusion, addition of Si to the culture medium promoted shoot regeneration of *A. multiflora *by altering activity of antioxidant enzymes. The inclusion of NaCl significantly decreased shoot regeneration and growth of *A. multiflora*. Addition of Si to the NaCl containing medium enhanced shoot regeneration and growth of *A. multiflora *by altering activity of antioxidant enzymes, maintenance of ultra structure of stomata, and limiting NaCl deposition in leaves. The present study can also be used for commercial extraction of active ingredients for pest management purposes.

## Figures and Tables

**Table 1:** Effect of 2-iP and NAA on adventitious shoot regeneration from leaf and petiole explants of *A. multiflora*.

Conc. (*µ*M)		Shoot induction (%)		No. of shoots/explant	
2-iP	IAA	Leaf	Petiole	Leaf	Petiole
0	0	0.0^d^	0.0^d^	0.0^d^	0.0^d^
4.4	5.7	41.2^c^	37.8^c^	2.6^c^	2.0^c^
8.8	5.7	50.6^b^	63.4^b^	4.8^b^	3.4^b^
12.2	5.7	73.3^a^	76.6^a^	8.5^a^	6.2^a^

Means within a column followed by the same letters are not significantly different (*P* ≤ 0.05).

**Table 2:** Effect of Si and NaCl on adventitious shoot regeneration from leaf and petiole explants of *A. multiflora*.

Conc. (mM)		Shoot induction (%)		No. of shoots/explant	
Si	NaCl	Leaf	Petiole	Leaf	Petiole
0	0	73.3^c^	76.6^d^	8.5^d^	6.2^d^
1.8	0	96.7^b^	94.6^b^	9.5^cd^	9.2^bc^
3.6	0	100^a^	100^a^	13.8^b^	10.8^b^
7.2	0	100^a^	100^a^	22.7^a^	18.4^a^
0	50	43.6^d^	31.2^e^	3.0^e^	1.7^e^
1.8	50	72.4^c^	91.3^c^	7.0^d^	6.4^d^
3.6	50	97.2^b^	98.6^ab^	8.3^d^	8.7^c^
7.2	50	100^a^	100^a^	10.7^c^	7.4^cd^

Means within a column followed by the same letters are not significantly different (*P* ≤ 0.05).

**Table 3:** Effect of Si and NaCl on growth characteristics of *A. multiflora*.

Conc. (mM)		Plant height (cm)	No. of leaves	Chlorophyll (*µ*g mg^−1^ fw)	Root length (cm)	Fresh weight (mg)		Dry weight (mg)	
Si	NaCl					Shoot	Root	Shoot	Root
0	0	8.1^b^	14.2^a^	3.04^c^	12.6^c^	1.28^f^	0.57^de^	0.14^c^	0.086^e^
1.8	0	8.6^a^	13.7^ab^	3.59^b^	13.0^bc^	1.52^d^	0.84^b^	0.17^b^	0.115^b^
3.6	0	8.7^a^	13.4^ab^	3.75^a^	13.6^c^	1.75^b^	0.89^a^	0.19^a^	0.135^a^
7.2	0	7.4^bc^	12.1^b^	2.83^d^	15.4^a^	1.70^c^	0.71^c^	0.18^ab^	0.094^d^
0	50	4.5^f^	8.3^d^	0.73^g^	7.06^d^	1.09^g^	0.36^f^	0.12^d^	0.054^f^
1.8	50	5.3^d^	12.6^b^	2.38^e^	14.8^b^	1.73^b^	0.59^d^	0.17^b^	0.095^d^
3.6	50	6.5^c^	12.8^b^	3.73^a^	15.8^a^	1.84^a^	0.60^d^	0.18^ab^	0.102^c^
7.2	50	5.2^d^	10.5^c^	1.66^f^	14.7^b^	1.36^e^	0.53^e^	0.17^b^	0.091^de^

Means within a column followed by the same letters are not significantly different (*P* ≤ 0.05).

**Table 4:** Effect of Si on antioxidant enzyme activities (U mg^−1^ protein) in regenerated shoot buds of *A. multiflora*.

Si (mM)	SOD	POD	APX	CAT
0	0.093^d^	1.28^c^	17.14^c^	1.62^c^
1.8	0.113^c^	1.38^c^	17.43^c^	1.87^bc^
3.6	0.126^b^	2.66^b^	26.08^a^	2.56^a^
7.2	0.133^a^	3.33^a^	19.41^b^	1.99^b^

Data represent mean of six independent measurements.

Means within a column followed by the same letters are not significantly different (*P* ≤ 0.05).

**Table 5:** Effect of Si and NaCl on antioxidant enzyme activities of *A. multiflora*.

Conc. (mM)		Leaf (U mg^−1^ protein)				Root (U mg^−1^ protein)			
Si	NaCl	SOD	POD	APX	CAT	SOD	POD	APX	CAT
0	0	0.030^c^	1.99^a^	3.33^e^	0.95^f^	0.041^d^	2.03^bc^	5.54^e^	1.91^c^
1.8	0	0.032^bc^	1.42^b^	4.01^d^	0.71^g^	0.047^c^	2.44^b^	5.41^e^	2.15^b^
3.6	0	0.039^a^	1.10^c^	4.24^cd^	1.29^e^	0.050^b^	2.78^a^	5.92^d^	2.20^b^
7.2	0	0.035^b^	0.72^d^	4.84^c^	1.41^d^	0.045^c^	2.86^a^	6.11^cd^	2.39^b^
0	50	0.013^e^	2.78^a^	6.63^a^	2.93^a^	0.028^e^	2.13^bc^	8.90^a^	3.76^a^
1.8	50	0.026^d^	2.57^b^	6.42^a^	2.76^b^	0.044^cd^	2.41^b^	7.33^b^	3.31^b^
3.6	50	0.033^bc^	2.15^c^	5.82^b^	1.69^c^	0.049^b^	2.70^a^	6.62^c^	3.09^bc^
7.2	50	0.028^cd^	1.75^de^	5.90^b^	1.44^d^	0.058^a^	1.82^c^	4.44^f^	2.83^d^

Data represent mean of six independent measurements.

Means within a column followed by the same letters are not significantly different (*P* ≤ 0.05).
